# The effect of informing patients with video before cardiac surgery on intensive care experience: A randomized controlled trial

**DOI:** 10.12669/pjms.40.6.8627

**Published:** 2024-07

**Authors:** Alev Kalkan, Figen Digin

**Affiliations:** 1 Alev Kalkan, BSN, MSc Nurse, Gebze Fatih State Hospital, Cardiovascular Surgery Intensive Care Unit, Turkey; 2 Figen Digin, BSN, MSc, PhD. Associate Professor, Department of Surgical Nursing, Kirklareli University Faculty of Health Sciences, Kirklareli, Turkey

**Keywords:** Cardiac surgery, Intensive care unit, Patient experience, Video

## Abstract

**Objectives:**

To study the effect of informing patients with video before cardiac surgery on intensive care experience.

**Methods:**

This randomized controlled trial was conducted between December 2021 and December 2022 in the cardiovascular surgery clinic of a public hospital with the participation of 90 patients (45 patients in experimental group - 45 patients in control group) who were scheduled to undergo cardiac surgery. Patient Information Form and Intensive Care Experiences Scale were used for study data. Patients in experimental group were informed with video about the intensive care before cardiac surgery.

**Results:**

It was found that the total score on ICES of the experimental group (74.5±3.9) was statistically and significantly higher than that of the control group (63.9±6.4) (p<0.001). The sub-dimension of awareness of surroundings (20.8±1.7), the frightening experiences (18.6±1.0), and the recall of experience (18.5±1.5) and satisfaction with care (16.7±1.4) were found to be statistically significantly higher in the experimental group, than in the control group sub-dimension scores (p<0.001).

**Conclusion:**

It was found that informing patients with video about the intensive care setting and process before cardiac surgery had a positive effect on the intensive care experience.

***Note:*** The study was produced from a master’s thesis and was not presented. All participants gave informed consent for the study, and that their anonymity was preserved.

**Trial Registration NO.:** NCT05255887.

## INTRODUCTION

Postoperative follow-up, treatment, and care processes of patients who undergo cardiac surgery are performed in intensive care unit (ICU).^1^ The period affecting the survival rates in most cardiac surgeries most is the early postoperative period, which is spent in the ICU.^2^ Nursing care and patient training play important roles in reducing the problems that may occur in the intensive care process after cardiac surgeries.^3^

ICU, which is a serious source of anxiety, cause great psychological and social burdens on individuals.^4^ Psychological and sociological problems faced by patients in ICU cause negative experiences.^5^,^6^ Life-threatening disease, presence of endotracheal tube, pain, thirst, fear, anxiety, sleep disorders, nightmares, and hallucinations are among the most commonly reported stressors in intensive care patients.^7^ Also, patients do not know that ICU is isolated and closed settings.^8^ The uncertainty in ICU increases surgical anxiety and stress in patients and affects surgical process and the intensive care process.^9^ Negative experiences in ICU may also prolong the recovery and discharge times of patients.^10^ The purpose of the intensive care team is to ensure that the patients are discharged from ICU with healing and positive experiences.^11^

The process of informing the patients before the surgical procedure must include the introduction of the surgery team, details of the intensive care setting, pain control methods, nutrition, presence of tubes and drains, lying position, and mobilization. Patients who are informed about the possible problems they may encounter after the surgery and the treatment process can have a better intensive care experience with the information given.^9^,^12^ Kalogianni et al.^13^ reported that pre-operative training given by nurses reduces anxiety and complications in patients undergoing cardiac surgery. Gökçe et al. 14 reported that training given by nurses reduces anxiety levels and positively affects physiological variables. For this reason, it is considered that informing patients about intensive care in the preoperative period would have an impact on their postoperative intensive care experience.

Verbal information, written brochures and multimedia supported (video, CD-ROM or internet) current training methods are used to inform patients. Video information, which is preferred in patient training and information processes in recent years, has become even more effective with the development of audio-visual technology. The use of videos in patient training is effective in helping patients learn more about the treatment process and better adapt to treatment.^15^ In previous studies, it was reported that informing with video is an effective and useful method in reducing the anxiety levels of patients.^16^

The purpose of the study was to evaluate the effect of informing patients with video before cardiac surgery on intensive care experience. Study Hypotheses was that Informing patients with a video before cardiac surgery has an effect on their intensive care experience.

## METHODS

The randomized controlled trial was conducted between December 2021 and December 2022 in the cardiovascular surgery clinic of a public hospital. The population of the study consisted of those who were hospitalized in the cardiovascular surgery clinic and who were scheduled to undergo cardiac surgery. The sample of the study consisted of patients who volunteered to participate in the study. The average ICES score in Kavuncu et al. study^1^ was determined to be 57.075.6. A total of 45 patients were recruited for each group, and 90 patients were included in the sample to test the 0.6-unit effect size value that was determined by accepting a 5% difference from this score in the experimental group as clinically significant with a 5% margin of error, a 95% confidence interval, and 80% power value. A randomization plan was prepared for a total of 90 patients by using a probabilistic scheme-based computer program to ensure randomization. The researcher directed the randomization. Patients who volunteered to participate in the study accepted randomization and were able to communicate in Turkish, had cognitive competence, were open to communication and cooperation, and were scheduled to undergo cardiac surgery were included in the sample. Those who were scheduled for emergency surgery were not included in the study. The study was completed with 90 patients.

### Data Collection Tools

Patient Information Form and Intensive Care Experiences Scale were used for research data.

### Patient Information Form

There were 12 questions (age, length of stay in ICU, gender, education status, working status, marital status, residence, income, social security, chronic disease, surgery experience, ICU experience) in the form prepared by the researcher.

### Intensive Care Experiences Scale (ICES)

The ICES was created in 2004 by Rattray et al.^9^, and its Turkish reliability and validity assessments were completed by Demir et al.^5^ in 2009. The person can assess his or her experiences in ICU using 19 scaled items. Scores on the ICES range from 19 to 95. The scale’s questions 7, 8, 9, 10, 15, and 17 are scored in reverse. There are four sub-dimensions on the scale: “Awareness of surroundings,” “Frightening experiences,” “Recall of experience,” and “Satisfaction with care.” The patient’s experience is rated as pleasant and his or her awareness as high as “he scale’s total score indicates”. The Cronbach alpha value of the Turkish translation of ICES was 0.79, and for this study it was 0.77.

### Blinded

The study’s researcher was aware of the patients who would get the video method of information. The researcher and the patients who were told before the surgery by video technique could not be blinded due to the nature of the study. The person performing the statistical evaluation was blind to the study data.

### Data Collection

The goal, significance, and context of the study were verbally explained to the patients admitted for cardiac surgery. A computer program based on probabilistic order was used to provide randomization of the patients and patients were divided into experimental and control groups.

Patient Information Form was filled by researcher in patient room of cardiovascular surgery clinic 24-48 hours before surgery for all the patient. No attempt was made to the control group other than the routine surgical preparation (legal preparation, physical preparation, psychological preparation) before the surgery.

As well as the routine surgery preparation, experimental group patients watched the informational video in a single room. The video lasted 9.08 minutes and was prepared by the researcher, who is a cardiovascular surgery intensive care nurse, with the support of the literature data. The average surgery time, the transition to ICU, the physical setting of ICU, the respiratory device, intubation and extubation, medical devices, breathing and coughing exercises, use of pillows, triflow and compression stockings, the actions that must and must not be done to protect the sternum, sleeping, food and liquid consumption, treatments, pain management, personal care and how to meet needs, information about each treatment were included in the content of the video. The video was shot over the scenario with the volunteer clinical nurse. An academic nurse, a clinical nurse, and a cardiac surgeon were consulted for the video. The video was shown to patients with a flash memory stick, using the television in room. The patients watched the video in a comfortable position sitting on bed or armchair. During the video, researcher was with patients and repeated viewings were made when needed. Patients’ questions were answered by the researcher using communication triangle, either by stopping while watching video or collectively at the end of the video.

**Fig.1:**
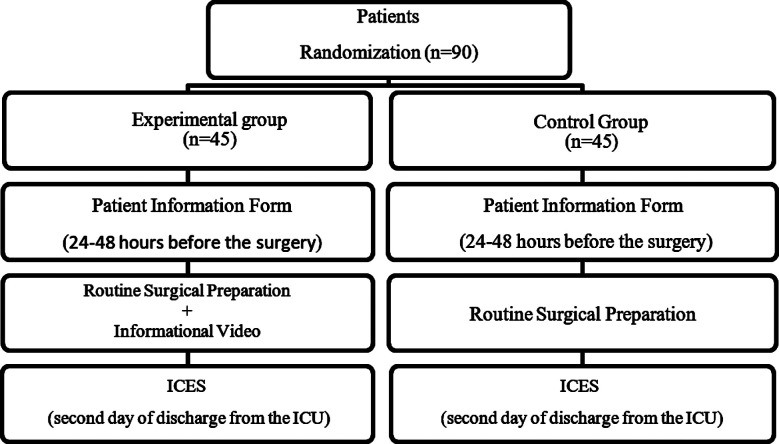
Flow Chart.

The day of discharge from the ICU was counted as the first day, and on the second day, the ICES was applied to all the patients by the researcher in patient room of the cardiovascular surgery clinic with face-to-face interview method (Fig.1). It took an average of 15 minutes to fill out the data collection tools, and an average of 20 minutes to inform the experimental group with the video.

### Ethical considerations

ClinicalTrials.gov has the study listed as NCT05255887. During the course of the investigation, Declaration of Helsinki and Good Clinical Practice Guidelines were taken into consideration. The Ethics Committee (2021/PR0365R01 – 12/28) as well as the administration of the university hospital gave their written consent. Patients were informed about the study prior to it starting, and written informed permission was obtained. Volunteer clinical nurse playing the patient role in the video provided written and verbal informed consent.

### Data Evaluation

Mean ± standard deviation was used as descriptive statistics for quantitative data, and numbers and percentages values were used as descriptive statistics for categorical data. The conformity of the quantitative data to the normal distribution was examined with the Shapiro-Wilk Test. The Student’s t-test was used to compare the normally distributed age variables between the groups, and the Mann-Whitney U Test was used to compare the non-normally distributed ICU length of stay and ICES scores. The Chi-Square Test was used to compare the categorical data between the groups. The statistical package program SPSS 20.0 was used to analyze the data. A value of p<0.05 was accepted as statistical significance.

## RESULTS

The mean age of the patients was 61.5±10.0 in experimental group and 60.8±9.7 years in control group (p=0.689). The length of stay in the ICU of patients in experimental group was 2.1±2.5 days, the length of stay in the ICU of the patients in control group was 2.0±1.7 days, and no significant differences were detected between the patients (p=0.487). When the experimental and control groups were compared according to categorical socio-demographic characteristics, no significant difference was found between the groups (p> 0.05), (Table-I).

**Table-I:** The Comparison of the Socio-Demographic Characteristics of the Patients.

		Experimental (n=45)	Control (n=45)	p
Age		61.5±10.0	60.8±9.7	0.689 ^a^
Length of stay in ICU (day)		2.1±2.5	2.0±1.7	0.487^b^
Gender	Female	7 (15.6)	15 (33.3)	0.086 ^c^
	Male	38 (84.4)	30 (66.7)	
Education status	Primary school	23 (51.1)	29 (64.4)	0.425 ^d^
	High school	16 (35.6)	11 (24.4)	
	University	6 (13.3)	5 (11.1)	
Working status	Working	23 (51.1)	18 (40.0)	0.397 ^c^
	Not working	22 (48.9)	27 (60.0)	
Marital status	Married	38 (84.4)	35 (77.8)	0.590 ^c^
	Single	7 (15.6)	10 (22.2)	
Place of residence	City	3 (6.7)	4 (8.9)	0.342 ^d^
	District	41 (91.1)	37 (82.2)	
	Village/Town	1 (2.2)	4 (8.9)	
Income	Income less than expenses	15 (33.3)	19 (42.2)	0.639 ^d^
	Income equals expense	23 (51.1)	21 (46.7)	
	Income more than expenses	7 (15.6)	5 (11.1)	
Social security	Yes	45 (100)	45 (100)	-
	No	0 (0)	0 (0)	
Chronic disease	Yes	38 (84.4)	35 (77.8)	0.590 ^c^
	No	7 (15.6)	10 (22.2)	
Surgery experience	Yes	15 (33.3)	24 (53.3)	0.089 ^c^
	No	30 (66.7)	21 (46.7)	
ICU experience	Yes	33 (73.3)	39 (86.7)	0.188 ^c^
	No	12 (26.7)	6 (13.3)	

a Student’s t-test,

b Mann Whitney U-test,

c Yates Chi-Square Test,

d Pearson Chi-Square Test.

It was found that the total score on ICES of the experimental group (74.5±3.9) was significantly higher than control group (63.9±6.4) (p<0.001). Experimental group’s the sub-dimension of awareness of surroundings (20.8±1.7), the frightening experiences (18.6±1.0), and the recall of experience (18.5±1.5) and satisfaction with care (16.7±1.4) were found to be significantly higher than the control group (p<0.001) (Table-II).

**Table-II:** The Comparison of the ICES Scores of the Patients.

	Experimental (n=45)	Control (n=45)	p*
ICES Total Score	74.5±3.9	63.9±6.4	<0.001
Awareness of surroundings	20.8±1.7	18.7±2.3	<0.001
Frightening experiences	18.6±1.0	16.0±1.7	<0.001
Recall of experience	18.5±1.5	16.6±3.3	<0.001
Satisfaction with care	16.7±1.4	12.7±1.8	<0.001

* Mann Whitney U-test.

## DISCUSSION

In study that the mean total score of the experimental group patients in ICES and sub-dimensions in terms of awareness of surroundings, frightening experiences, recall of experience and satisfaction with care were significantly higher than control group.

Sorlie et al.^17^ reported that when patients who underwent cardiac surgery were informed by video by nurses after admission to the clinic in the preoperative period, the patients had lower levels of anxiety and their subjective health status was better in the postoperative period. In his study, Pager et al.^18^ reported that patients who were informed by videos experienced significantly less anxiety, were more satisfied, perceived the procedure better, and felt better than the control group. Oliveira et al.^19^ reported that the use of video was effective in-patient education in his study conducted with patients who had coronary artery bypass surgery. In their randomized controlled study, Fleischer et al.^20^ found that a structured information program intended for patients in the ICU could reduce the anxiety experienced during their stay in the ICU. Lai et al.^21^ determined in their randomized controlled study that providing comprehensive preoperative information about the ICU setting increased patient and family satisfaction levels, and reduced patients’ anxiety levels. Kavuncu et al.^1^ reported that patients receiving information about the intensive care process before being admitted to the ICU had positive effects on satisfaction. Pazar et al. and Iyigun et al.^22^ reported that patients who were given training before cardiac surgery had higher patient-ventilator synchronization and lower anxiety levels in mechanical ventilation. Özer et al. and Akyil et al.^23^ examined the effects of providing information about the physical and technological setting of the ICU on the discomfort of patients during intensive care experiences and determined that the level of discomfort was low in the trained group. A study addressing patient experiences to improve symptom management and experience of intensive care patients reveals that intensive care patients have information needs.^24^ According to the research results, informing patients with video positively affects patient outcomes.

When compared to conventional orientation, Oliveira et al.^19^ showed that video sources were more successful at boosting patient knowledge during preoperative orientation for cardiac surgery. In their study conducted to evaluate the effects of video information on anxiety, stress, and depression of patients undergoing coronary angiography, Jamshidi et al.^16^ determined that using video information method in patient education is a useful method in reducing the psychological parameters of patients undergoing coronary angiography.

### Limitations

It is single-centered. Multicenter randomized controlled studies are needed to confirm the research findings. Due to individual and treatment protocol differences, ensuring homogeneity may add to the limitations.

## CONCLUSION

It was found that informing patients with video before cardiac surgery had a positive effect on the intensive care experience. It was also found that the patients who were informed by video were aware of the setting in ICU, were aware of the pessimistic experiences, remembered the experiences, and were satisfied with the care received in ICU. Instead of standard training methods, it must be supported to provide training about intensive care setting by using multimedia-supported, visual and auditory materials.

### Recommendations:

We recommend that video information be used by surgical nurses at different stages of care and in different patient groups.

### Authors’ Contribution:

**AK**: Conceived, designed manuscript, data collection, manuscript writing, literature review, statistical analysis.

**FD**: Conceived, designed manuscript, manuscript writing, literature review, statistical analysis, critical discussion, did editing and finally approved manuscript, responsible and accountable for the accuracy and integrity of the work.
